# Macrophages expressing TREM-1 are involved in the progression of HPV16-related oropharyngeal squamous cell carcinoma

**DOI:** 10.1080/07853890.2021.1905872

**Published:** 2021-03-26

**Authors:** Barbara Azzimonti, Luca Raimondo, Diletta Francesca Squarzanti, Tiziana Rosso, Paola Zanetta, Paolo Aluffi Valletti, Luigi Chiusa, Laura Masini, Giancarlo Pecorari, Mario Airoldi, Marco Krengli, Mirella Giovarelli, Guido Valente

**Affiliations:** a Center for Translational Research on Autoimmune and Allergic Diseases (CAAD), Department of Health Sciences (DiSS), University of Piemonte Orientale (UPO), Novara, Italy; b Otorhinolaryngology Division, Department of Surgical Sciences, University of Turin, Turin, Italy; c Clinical Epidemiology Unit, “Città della Salute e della Scienza” Hospital – CPO Piemonte, Torino, Italy; d Division of Ear Nose and Throat Department-Head and Neck Surgery, DiSS, University of Piemonte Orientale (UPO), Novara, Italy; e Department of Biomedical Sciences and Human Oncology, University of Turin, Turin, Italy; f Radiotherapy Unit, Department of Translational Medicine (DiMeT), University of Piemonte Orientale (UPO), Novara, Italy; g Department of Oncology, Azienda Ospedaliera Universitaria Citta’ della Salute e della Scienza di Torino, Turin, Italy; h Department of Molecular Biotechnology and Health Sciences, University of Turin, Turin, Italy; i Center for Experimental Research and Medical Studies (CERMS), AOU Città della Salute e della Scienza di Torino, Turin, Italy; j Pathology Unit, Ospedale “Sant’Andrea”, DiMeT, University of Piemonte Orientale (UPO), Vercelli, Italy

**Keywords:** Human papillomavirus (HPV), oropharyngeal squamous cell carcinoma (OP-SCCs), tumoral microenvironment (TME), peritumoral and intratumoral infiltration, triggering receptor expressed on myeloid cells-1 (TREM-1)

## Abstract

**Introduction:**

Many types of research have been performed to improve the diagnosis, therapy, and prognosis of oropharyngeal carcinomas (OP-SCCs). Since they arise in lymphoid-rich areas and intense lymphocytic infiltration has been related to a better prognosis, a TREM-1 putative function in tumour progression and survival has been hypothesized.

**Materials and methods:**

Twenty-seven human papillomavirus (HPV) 16^+^ OP-SCC specimens have been analyzed to relate TREM-1 expression with histiocytic and lymphocytic markers, HPV presence and patients’ outcome.

**Results:**

No differences have been shown between intratumoral and stromal CD4^+^ cells, while intratumoral CD8^+^ lymphocytes are higher with respect to the tumour stroma (*p*** **=** **.0005). CD68^+^ cells are more than CD35^+^ and TREM-1^+^; their presence is related to CD35^±^ and TREM-1^±^ histiocytes (*p*** **=** **.005 and .026, respectively). Intratumoral CD4^+^ lymphocytes are higher in p16^+^ cases (11/27) than in p16^−^ (*p*** **=** **.042); moreover, p16 positivity correlates to a better survival (*p*** **=** **.034). CD4^+^, CD8^+^ and CD35^+^ cells have no impact on survival, while CD68 expression heavily influences progression and bad outcome (*p*** **=** **.037). TREM-1 positivity also leads to worst overall survival (*p*** **=** **.001): peritumoral expression and death-cause relationship are always significant, particularly when the cause is OP-SCC (*p*** **=** **.000).

**Conclusion:**

While p16 shows to better stratify HPV16^+^ patients’ outcome, TREM-1^+^ macrophages suggest their key importance in HPV-related OP-SCCs progression.

**KEY MESSAGES**
- TREM-1 positivity correlates to the worst overall survival of HPV16-positive OPSCCs-affected patients.
- p16-positive HPV16 related OPSCCs patients have a better prognosis with respect to p16-negative ones.

## Introduction

In the last 10 years, many advances have been fulfilled on the natural history, diagnosis, therapy, and prognosis of oropharyngeal squamous cell carcinomas (OP-SCCs).

A crucial point is a distinction between human papillomavirus (HPV)-related and non-related tumours, conventionally made up by p16 immunohistochemical positivity, if adequately evaluated [1,2], since the first group of affected patients is characterized by a better prognosis, in terms of disease-free and overall survival (OS).

Routine histology has demonstrated that HPV-related carcinomas show peculiar findings, for instance, non-keratinizing, basaloid, papillary and lymphoepithelial patterns [3–6].

Moreover, much interest has been devoted to the cellular components of the tumoral microenvironment (TME) and their functional implications: for instance, the presence of an intense lymphocytic infiltration is associated with a better prognosis [6]; the qualitative analysis, made by lymphocytic immunophenotyping, has demonstrated that high amounts of peritumoral and intratumoral CD8^+^ cells correspond to a significant-good prognosis in HPV-related tumours [7–9]; CD4^+^ lymphocytes are also significantly more numerous in HPV-tumours, while T regulatory lymphocytes CD4^+^ FoxP3^+^ are not differently represented in HPV and non-HPV tumours [10]. Independently from the HPV association, natural killer CD56^+^ lymphocytes are consistently more represented in cases with better outcomes.

In the effort to determine the conditions for the therapy improvement, the expression of PD-L1, ligand for the receptor PD-1 [11] has been also made up, both on tumour cells and in infiltrating lymphocytes: in some recent studies, high amounts of PD-L1^+^ lymphocytes predict a better prognosis, whilst tumour cells positivity seems not to be significant [9,12]. In the framework made up of cells and stromal tissues surrounding the tumours, the macrophages seem to play a not negligible role: CD68^+^ macrophages are known to be present in HPV-tumours more than in negative ones; however these cells express PD-L1 mainly in HPV-negative cases [13].

This observation has induced us to evaluate selective populations of histiocytic cells in the OP-SCCs and to determine the presence of their distinctive subclasses in relation to the patients’ outcome. In particular, TREM-1, a protein of the group Triggering Receptor Expressed on Myeloid cells, present in neutrophils and monocytes [14], has been shown to participate in different pathological conditions, including inflammatory and autoimmune diseases, atherosclerosis and malignant tumours [15,16]. In all studies conducted up to now on tumour series, TREM-1 proved to be associated to aggressive courses or prognosis, perhaps through its expression on dendritic cells [17]: its negative prognostic role was observed in non-small cell lung cancer, by measuring the molecule in pleural effusions [15]; the expression levels of TREM-1 gene are more higher in primary and metastatic colon carcinomas than in colonic normal mucosa [18]; aggressiveness of hepatocellular carcinoma seems to be also affected by TREM-1 immunohistochemical expression on stellate cells [19,20].

Because of its onset in a lymphoid-rich area, oropharyngeal carcinoma may be a promising model to evaluate the putative role of TREM-1 in the progression and prognosis of the tumour. On this basis, we have analyzed a series of surgical specimens of OP-SCC, to relate the expression of TREM-1 with other histiocytic and lymphocytic markers, HPV status, p16 positivity and patients’ outcome.

## Materials and methods

### Patients

The series included 27 surgical specimens (1 well-differentiated G1, 12 moderately differentiated G2 and 14 poorly differentiated G3 lesions) retrospectively selected from patients with a bioptic diagnosis of HPV16-positive OP-SCCs, treated in the period 2004–2017. The cases were enrolled from the archives of three University Italian Units of Pathology: Torino (Città della Salute Hospital), Novara (Maggiore della Carità University Hospital) and Vercelli (Sant’Andrea Hospital). The main subsites of the tumours were tonsil, tonsillar pillars and soft palate. Patients with other synchronous or metachronous head and neck carcinomas, cured with neo-adjuvant therapy, previously treated for other tumours, with bony invasion or immunosuppressed after organ transplantation, were excluded.

HPV16 presence, molecularly assessed with the E6HPV16 primers couple (F: 5′-GAG AAC TGC AAT GTT TCA G-3′ and R: 5′-GAT GAT CTG CAA CAA GAC-3′), was also detected via p16 immunohistostaining.

Exposure risk factors for OP-SCCs, like smoking and alcohol consumption [21], were also registered in the analysis. Patients’ characteristics, cause of death and tumour staging were collected from the clinical records and informatic systems of the afore-mentioned hospitals (Table 1).

**Table 1.** Characteristics of patients and oropharyngeal neoplasms.

	OP-SCC		
	G1	G2	G3
Patients, *n*	1	12	14
Sex, *n* (%)			
Male	1 (100.00)	9 (75.00)	11 (78.57)
Female	0 (0.00)	3 (25.00)	3 (21.43)
Age (years), median (IQR)	43 (43-43)	59.5 (52.5-66)	64 (62-71)
Staging, *n* (%)			
3	1 (100.00)	8 (66.67)	6 (42.86)
4a	0 (0.00)	4 (33.33)	8 (57.14)
Tobacco consumption, *n* (%)			
No	0 (0.00)	3 (25.00)	3 (21.43)
Light smoker	1 (100.00)	1 (8.33)	2 (14.29)
Medium smoker	0 (0.00)	0 (0.00)	5 (35.71)
Heavy smoker	0 (0.00)	7 (58.34)	3 (21.43)
Missing	0 (0.00)	1 (8.33)	1 (7.14)
Alcohol consumption, *n* (%)			
No	0 (0.00)	4 (33.33)	1 (7.14)
Low risk	1 (100.00)	2 (16.67)	4 (28.57)
High risk	0 (0.00)	2 (16.67)	4 (28.57)
Harmful	0 (0.00)	3 (25.00)	4 (28.57)
Missing	0 (0.00)	1 (8.33)	1 (7.14)
Cause of death, *n* (%)			
Tumour persistence	0 (0.00)	2 (16.67)	2 (14.29)
Tumour relapse	0 (0.00)	2 (16.67)	2 (14.29)
Other tumours	0 (0.00)	2 (16.67)	0 (0.00)
Other causes	0 (0.00)	1 (8.33)	2 (14.29)
Alive	1 (100.00)	5 (41.67)	8 (57.14)

OP-SCC, oropharyngeal squamous cell carcinoma; G1, well-differentiated; G2, moderately differentiated; G3, poorly differentiated OP-SCCs; IQR, interquartile range.

All subjects gave informed consent to participate in the *ex vivo* immunohistochemical study, conducted in an ethical and responsible manner, according to the Helsinki Declaration guidelines of 1975, revised in 2013; patients’ data were all fully anonymized.

### Histology

All slides, routinely stained by haematoxylin-eosin (H-E), were revised by one of us (GV), to reformulate the diagnosis and the histologic grading.

### Immunohistochemistry

For each case, deparaffinized sections were stained by the following antibodies: cCDKN2A/p16INK4a (clone 2D9A12, working dilution 1:1000, Abcam); CD4 (clone 4B12, working dilution 1:50, Thermo Fisher Scientific); CD8 (clone C8/144B, working dilution 1:50, Thermo Fisher Scientific); CD35 (clone Ber-MAC-CDR, 1:40, DAKO); CD68 (clone KP1, working dilution 1:100, Thermo Fisher Scientific) and TREM-1 (clone 174031, working dilution 1:100, R&D System). Briefly, after appropriate unmasking and blocking of endogenous peroxidase activity, the sections were incubated with opportunely diluted antibodies using Ventana benchmark XT Automated Platform (Ventana Medical System); 3,3-diaminobenzidine (DAB, Bio-Optica) was used as the chromogen. Sections were counterstained by Mayer’s haematoxylin (05-06002, Bio-Optica) and then mounted with a permanent mounting medium. Negative controls were included by omitting the primary antibodies.

### Interpretation of immunohistochemical reactivity

All stained slides were captured by scanner through the Panoramic Viewer software (3D Histech, distributed by Diapath). The intensity and distribution patterns of the stainings were analyzed by two blinded, independent observers (GV and BA), with >90% concordance. When possible, the same fields were observed to limit the variability. For each case, ten random high-power fields (HPF x400), were selected. Positive and total cell number was recorded using Image-Pro Plus 6.0 software technology (Media Cybernetics).*p16 staining* – Each case was considered positive when more than 70% of neoplastic tissue showing strong and diffuse nuclear and cytoplasmic immunostaining.

*CD4 and CD8 staining* – Infiltration by CD4 and CD8 lymphocytes was evaluated separately inside the neoplastic sheets (intratumorally) and on the invasion front (peritumorally). The results were expressed as the mean of the number of cells per HPF; a range 0–3 was employed, as follows: 0, no or very few cells; 1, <10% positive lymphocytes per HPF; 2, 10–30% lymphocytes per HPF; 3, >30% lymphocytes per HPF.

*TREM-1, CD35 and CD68 and staining* – A semiquantitative evaluation of these non-lymphoid cells was made on the invasion front and expressed as a mean of the number of reactive cells per HPF; a range 0–3 was employed differentially for CD35 and TREM-1 on one hand (0, negative; 1, few cells per HPF; 2, up to 10 cells per HPF; 3, > 10 cells for HPF) and for CD68 (0, no cells or few cells; 1, up to 20 cells per HPF; 2, 20–50 cells per HPF; 3, >50 cells per HPF) on the other.

### Statistical analysis

The distribution of the patients’ characteristics was summarized using frequency and percentage for qualitative variables, and median and interquartile range for continuous variables.

A non-parametric Wilcoxon matched-pairs signed-ranks test was used to verify the existence of differences in matched pairs.

The Chi-square test was used to study the relationships among the expression of the different markers (absence or presence) and between the markers and the clinical-pathological information (stage, the total and specific cause of death for OP-SCCs).

OS was estimated with the Kaplan–Meier method. The cumulative incidence of death was calculated from the date of diagnosis of OP-SCCs to the date of death, or the completion of follow-up. The cause-specific cumulative incidence function was estimated using the method proposed by Gooley et al. in presence of a competing event (death from other cause [22]). The Cox model was used to estimate the Hazard Ratios (HR) adjusted for the main prognostic factors. All statistical analyzes were performed by using the Stata version 15.1 software (Stata Corp, College Station). Statistical significance was set at *p*** **<** **.05.

### Statistical data

Statistical analysis data associated with this article can be found, in the online version, at DOI: 10.17632/v3yfzz5bhh.1.

## Results

### Patients

Characteristics of patients and neoplasms are depicted in Table 1. 21 of the 27 OP-SCC-affected subjects are males (77.8%) and 6 females (22.2%), with a median age of 63** **years (IQR 55–66).

Data regarding alcohol, tobacco consumption, sex, age and stage are stratified with respect to the tumour grade, together with patients’ cause of death due to tumour persistence/relapse, other tumour types or other causes.

### Immunohistochemical studies

#### Haematoxylin and eosin staining

**Figure 1.**
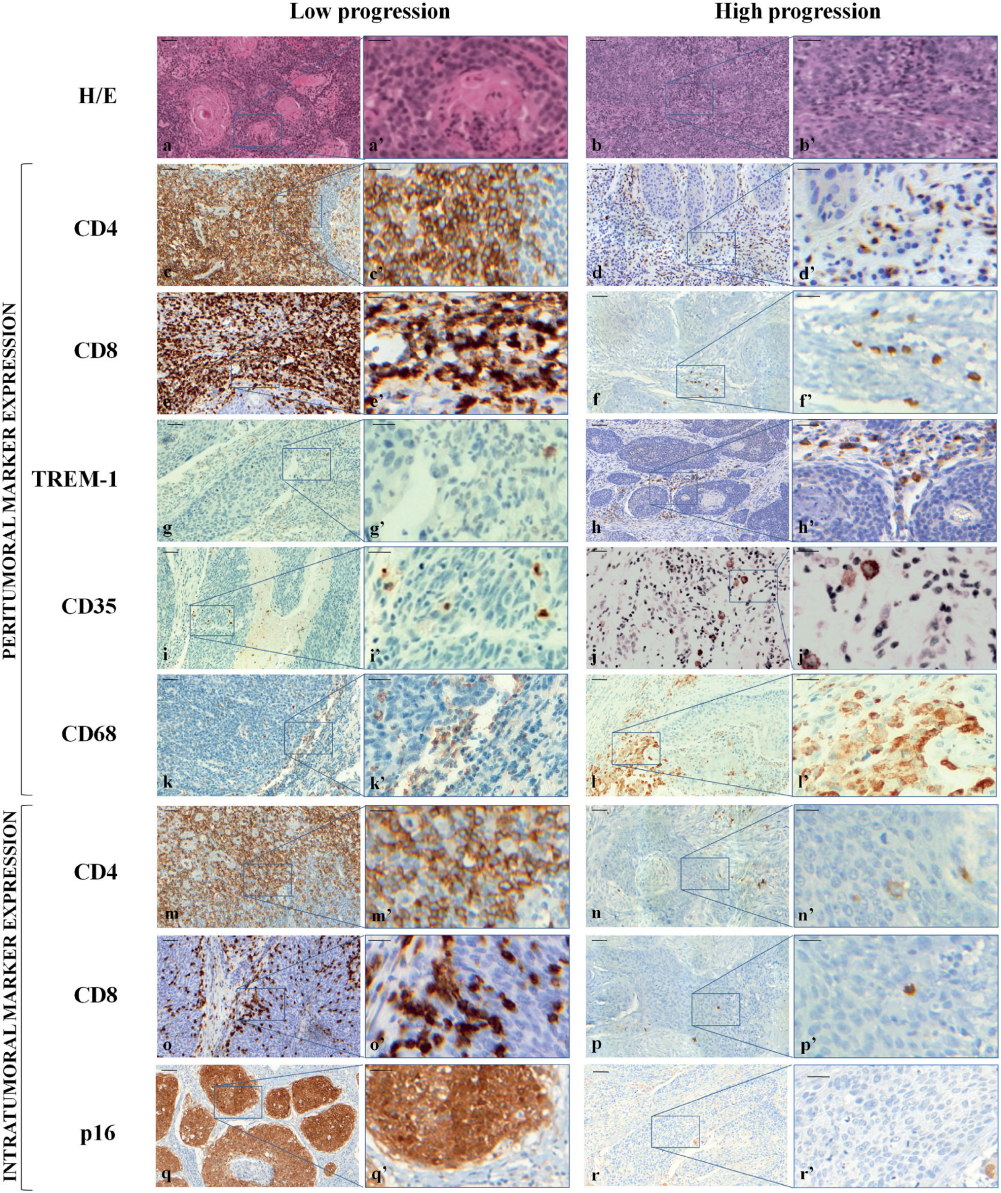
Representative haematoxylin and eosin (H-E) staining and expression of peritumoral and intratumoral markers in low and high progression OP-SCCs. Top panel: H-E staining. 20× (a, b) and 40× (a’, b’). Middle panel: peritumoral infiltration expression. CD4: 20× (c, d) and 40× (c’, d’); CD8: 20× (e, f) and 40× (e’, f’); TREM-1: 20× (g, h) and 40× (g’, h’); CD35: 20× (i, j) and 40× (i’, j’); CD68: 20× (k, l) and 40× (k’, l’). Bottom panel: intratumoral markers expression. CD4: 20× (m, n) and 40× (m’, n’); CD8: 20× (o, p) and 40× (o’, p’); p16: 20× (q, r) and 40× (q’, r’). The black boxes in the 20× fields correspond to the 40× magnification fields. Markers have been developed with 3,3’-diaminobenzidine. Scale bar: in the 20× fields,50** **µm; in the 40× fields, 20** **µm.

Haematoxylin-eosin (H-E) staining of the histologically diagnosed OP-SCCs with diverse grading is depicted in Figure 1; representative cases at low (a, a’) and high (b, b’) tumour progression are shown.

#### Peritumoral and intratumoral lymphocytic and histiocytic infiltration

Peritumoral and intratumoral biological markers expression, detected by the immunohistochemical interpretation of the staining intensity and distribution patterns, is displayed in Figure 1 following the criteria described before. The percentage distribution of the lymphocytic and histiocytic infiltration, reported in Table 2, evidences their different immunological activity within the two compartments in G1, G2 and G3 OP-SCCs.

**Table 2.** Percentage distribution of peritumoral and intratumoral infiltration.

	OP-SCC		
	G1	G2	G3
Peritumoral			
CD4^+^			
0	0 (0)	0 (0)	0 (0)
1^+^	0 (0)	1 (8.33)	0 (0)
2^+^	1 (100)	3 (25)	2 (14.28)
3^+^	0 (0)	8 (66.67)	12 (85.72)
CD8^+^			
0	0 (0)	0 (0)	0 (0)
1^+^	0 (0)	7 (58.33)	4 (28.58)
2^+^	1 (100)	3 (25)	8 (57.14)
3^+^	0 (0)	2 (16.67)	2 (14.28)
TREM-1			
0	0 (0)	2 (16.67)	3 (21.43)
1^+^	1 (100)	7 (58.33)	6 (42.86)
2^+^	0 (0)	1 (8.33)	3 (21.43)
3^+^	0 (0)	2 (16.67)	2 (14.28)
CD35^+^			
0	0 (0)	2 (16.67)	0 (0)
1^+^	1 (100)	8 (66.67)	14 (100)
2^+^	0 (0)	2 (16.67)	0 (0)
3^+^	0 (0)	0 (0)	0 (0)
CD68^+^			
0	0 (0)	0 (0)	0 (0)
1^+^	0 (0)	1 (8.33)	0 (0)
2^+^	0 (0)	5 (41.67)	5 (35.71)
3^+^	1 (100)	6 (50)	9 (64.29)
Intratumoral			
CD4^+^			
0	0 (0)	0 (0)	0 (0)
1^+^	0 (0)	1 (8.33)	1 (7.14)
2^+^	1 (100)	4 (33.33)	3 (21.43)
3^+^	0 (0)	7 (58.34)	10 (71.43)
CD8^+^			
0	0 (0)	0 (0)	0 (0)
1^+^	0 (0)	2 (16.67)	1 (7.14)
2^+^	1 (100)	6 (50)	3 (21.43)
3^+^	0 (0)	4 (33.33)	10 (71.43)
p16^+^			
0	1(100)	7 (58.33)	8 (57.14)
1^+^	0 (0)	5 (41.67)	6 (42.86)

*Note*. Values are *n* (%).

Regarding CD4^±^ and CD8^±^ lymphocytes, different results have been observed. For CD4 marker, a peritumoral score of 1 has been depicted in 1/27 (3.70%), 2 in 6/27 (22.22%) and 3 in 20/27 (74.07%) cases; an intratumoral score of 1 in 2 cases out of 27 (7.41%), 2 in 8/27 (29.63%) and 3 in 17/27 (62.96%). For CD8, a peritumoral score of 1 was evidenced in 11/27 (40.74%), 2 in 12/27 (44.44%) and 3 in 4/27 (14.81%); an intratumoral score of 1 in 3 cases out of 27 (11.11%), 2 in 10/27 (37.04%) and 3 in 14/27 (51.85%). The percentage distribution of CD4 and CD8 scores within each G1, G2 and G3 OP-SCCs category is displayed in Table 2.

A non-parametric Wilcoxon test has been performed for paired data to check for the existence of differences within the same marker (intra- and peri-tumour); precisely, no statistically significant differences among intratumoral and stromal CD4^±^ cells have been calculated (*p*** **=** **.248, Table 3).

**Table 3.** Association among the biological markers evaluated in OP-SCC samples.

Biological markers	CD4 intra	CD4 peri	CD8 intra	CD8 peri	p16 intra	TREM-1 peri	CD35 peri	CD68 peri
CD4 intra	1							
CD4 peri	*0.248*	1						
CD8 intra	0.408	0.104	1					
CD8 peri	0.964	0.335	***0.0005***	1				
p16 intra	**0.042**	0.636	0.594	0.773	1			
TREM-1 peri	0.260	0.488	0.854	0.176	0.219	1		
CD35 peri	0.889	0.799	0.947	0.545	0.468	0.653	1	
CD68 peri	0.607	0.715	0.676	0.677	0.573	**0.026**	**0.005**	1

Intra, intratumoral; peri, peritumoral.

Statistically significant values (*p*** **<** **.05), calculated with Chi-square test or Wilcoxon test (italic), are in bold.

Conversely, the CD8 marker shows a statistically significant difference, with intratumoral CD8^±^ lymphocytes more numerous respect to those infiltrating the tumour stroma (*p*** **=** **.0005, Table 3).

Since the intratumoral distribution of non-lymphocytic cells is quantitatively neglectable, only stromal TREM-1, CD35 and CD68 positive cells have been considered and reported (Figure 1). Their descriptive percentage distribution in G1, G2 and G3 cases is shown in Table 2. For TREM-1, a peritumoral score of 0 in 5/27 (18.51%), 1 in 14/27 (51.85%), 2 in 4/27 (14.81%) and 3 in 4/27 (14.81%) has been calculated. Regarding peritumoral CD35 antigen, a score of 0 in 2/27 (7.40%), 1 in 23/27 (85.19%), 2 in 2/27 (7.41%) and 3 in any of the cases has been observed.

Finally, CD68^±^ histiocytic cells are much more represented than CD35 and TREM-1; in fact, it is present with a score of 1 in 1/27 cases (3.70%), of 2 in 10/27 (37.04%) and of 3 in the remaining 16 cases (59.26%).

The presence of relationships amongst the lymphocytic and histiocytic cells has been investigated with a Chi-square test. The presence of very numerous CD68^±^ histiocytes in the tumour stroma directly relates to high infiltration by TREM-1^±^ and by CD35^±^ histiocytes (*p*** **=** **.026 and .005, respectively; Table 3).

Eleven out of the 27 HPV16 infected cases (40.74%) are positive for the p16 intratumoral marker strongly represented and diffusely distributed throughout the tumoral areas; 0 of them is G1, 5 are G2 and 6 G3 OP-SCCs (Table 2).

p16 positive cases show a greater intratumoral CD4^±^ lymphocytes infiltration than negative ones, demonstrating a significant correlation (*p*** **=** **.042, Table 3).

### Correlation between lymphocytic, histiocytic infiltration and both clinical outcome and pathologic parameters

By the same Chi-square test, we analyzed the presence of the relationships between each immunophenotypic marker and the outcome, defined by pathological staging and OS. Although in series cases with high variability of tumour extension have been included, infiltration of neoplastic tissue by lymphocytes and histiocytes (identified by the previously described markers) is not related to the pathological stage.

The analysis of the immunophenotypic markers shows that infiltration by CD4^±^ and CD8^±^ lymphocytes, both inside the tumour and in the stroma, has not any impact on survival (Table 4).

**Table 4.** Association among biological markers and clinical-pathological information of OP-SCC patients.

Biological markers	Staging	Cause of death	Death due to tumour
CD4 intra	0.417	0.484	0.206
CD4 peri	0.509	0.254	0.554
CD8 intra	0.817	0.406	0.297
CD8 peri	0.955	0.296	0.457
p16 intra	0.930	**0.034**	0.053
TREM-1 peri	0.596	**0.001**	**0.000**
CD35 peri	0.128	0.607	0.536
CD68 peri	0.280	0.127	**0.037**

OP-SCC, oropharyngeal squamous cell carcinoma; intra, intratumoral; peri, peritumoral.

Slightly insignificant and statistically significant values (*p*** **<** **.05), calculated with Chi square test, are underlined and bolded, respectively.

The same test shows statistically significant relationships between both p16 and TREM-1 and the cause of death (*p*** **=** **.034 and .001 respectively; Table 4), and between peritumoral TREM-1 and death due to tumour. Precisely, *p*-values indicate that the relationship between peritumoral TREM-1 and death is always significant; particularly, the significance is very strong when the cause is the OP-SCC (*p*** **=** **.000; Table 4).

p16 positivity correlates to a better survival; in fact, 11 out of 13 dead are p16-negative cases: 7 patients died for OP-SCC relapse or persistence, 2 because of another tumour and 2 from other causes. Therefore, the Chi-square test evidences that the relationship between p16 and death is statistically significant for any cause of death, while it is slightly insignificant when the cause is the OP-SCC (*p*** **=** **.053; Table 4).

CD35 infiltration is not significantly correlated to death from any cause (Table 4).

By contrast, histiocytic infiltration is much more significant, since the presence of high amounts of CD68-positive cells heavily influences the progression and the bad outcome of the tumours (*p*** **=** **.037; Table 4).

**Figure 2.**
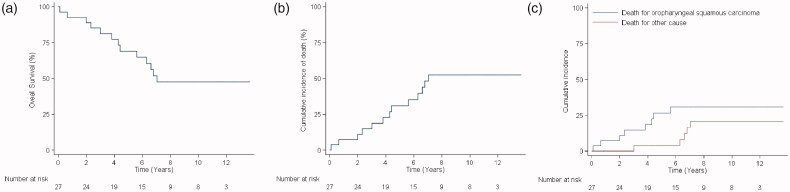
Graphical representation of (a) overall survival: the Kaplan–Meier method reveals a total 5-years overall survival of 51.85% (14 out of 27; 95% CI** **=** **47.5–83.3); (b) cumulative incidence of death from any cause: the 5-years cumulative incidence of death, calculated from the date of diagnosis to the date of death (end of follow-up), evidences that a total of 13 (48.15%) patients out of 27 died; (c) cumulative incidence of death stratified by cause: the 5-years cumulative incidence of death from oropharyngeal cancer (persistence and relapses) is 29.63% (8 out of 27; 95% CI** **=** **14.4–49.5), while death from other tumours and other causes is 18.52% (5 out of 27; 95% CI** **=** **0.7–28.1).

Overall, 14 patients out of 27 survived (Table 1 and Figure 2(a)). Therefore, the total 5-years OS calculated with the Kaplan–Meyer method is 69.1% (95% CI** **=** **47.5–83.3), while the cumulative incidence of death, calculated from the date of diagnosis to the date of death or end of follow-up, is 48.15% (Figure 2(a,b)), while the cumulative incidence of death, calculated from the date of diagnosis to the date of death or end of follow-up, is 30.9% (95% CI** **=** **16.8–52.5; Figure 2(a,b)).

In 8 out of 13 dead, OP-SCC persistence is the cause of death (2 G2 and 2 G3) or relapses (2 G2 and 2 G3); in the other 5 patients (3 G2 and 2 G3), the death is not related to the analyzed tumour type (Table 1 and Figure 2(c)). Therefore, the 5-years cumulative incidence of death from OP-SCC is 27.6% (95% CI** **=** **14.1–49.5), while that from other tumour types or causes is 4.6% (95% CI** **=** **0.7–28.1; Table 1 and Figure 2(c)).

## Discussion

In this study, a population of 27 patients, affected by a primitive HPV16 positive OP-SCC in an advanced stage and treated with homogeneous surgical and oncological criteria in three centres of the same geographical area, has been considered.

All tissue sections have been obtained from surgical specimens, accounting for a right extension of the neoplastic area, thus ensuring for an adequate evaluation of the immunophenotypical markers.

In fact, since distribution heterogeneity of the cells forming the background component of head and neck tumours is well demonstrated [23] and may distort its correct interpretation, we excluded from this series all cases represented by only bioptic fragments.

Furthermore, several studies and meta-analyses have identified p16 both as an effective surrogate marker of HPV infection [24] and as an indicator of better prognosis respect to HPV-negative cases [25,26]; for these reasons, HPV presence is usually validated by p16 antigen immunohistochemical overexpression. However, with this system, not only some HPV lesions may not be recognized as such, but also an accurate stratification of HPV-positive and p16-negative patients, with respect to disease progression and life expectancy, is prevented. Therefore, study cases have been selected on the basis of HPV16 presence, assessed by molecular PCR test; then a p16 immunostaining investigation has also been made. As revealed in our series, only 11 out of 27 HPV16-positive cases test also positive for p16, despite the use of the most recognized immunohistochemical reactivity interpretation method [27]. The combined identification system confirms the limitation of the immunohistochemical approach only and reveals that, with the same HPV infection, p16 negative cases have a faster and more aggressive disease course with respect to the positive ones, with the implications described next.

In the last few years, personalized therapies have been implemented to help to prevent and treat patients’ diseases. In tumours, all innate and adaptive immune cells are downregulated by specific signalling pathways that suppress their activity and effectiveness. For these reasons, much interest has been also devoted to the cellular components within the TME and their functional implications; therefore, we evaluated the expression of CD4, CD8, CD35, CD68 and TREM-1 markers.

In our work, CD4^±^ lymphocytes show to be highly represented both in the intratumoral and peritumoral environment, with CD4 infiltration more abundant in p16^±^ cases, confirming their contribution for a better outcome [28]. In fact, the key role of CD4^±^ cells is complex, since they help to coordinate the immune response, by stimulating other immune cells such as macrophages, B and CD8^±^ T lymphocytes. The presence of CD8^±^ cytotoxic T cell, lower than that of CD4 lymphocytes, is also important, since they cooperate in prognosis improvement in HPV-positive OPSCCs [9], because of their ability to kill transformed cancer cells.

As observed by Kitamura et al., CD68^±^ tumour-associated macrophages (TAMs), seem to play a not negligible role in this context [29]. In accordance with this, our data account for a negative role of TAMs on the survival of patients with OP-SCCs, by considering selectively the cells distributed in the perineoplastic stromal tissue. CD68 is a pan-macrophage marker, encompassing both the functional M1 (pro-inflammatory and anti-tumour) and M2 (anti-inflammatory and pro-tumour) profiles. The association of infiltrating macrophages with a poor prognosis, demonstrated by CD68 and/or CD163 immunophenotyping, has been previously reported in a number of head and neck tumours [30–34], but infrequently in selective series of OP-SCCs. The peculiar anatomic and immunologic characteristics of this subsite and the interference of HPV infection suggest that macrophages may sustain a stringent biologic significance.

For these reasons, we also investigated the distribution and the significance of subclasses of macrophages, in particular those of CD35 dendritic cells and not only those regarding TREM-1 expressing cells. Despite TREM-1 macrophages distributed outside show a strong and significant association with the OS, dendritic cells are rarely represented in the tumours and do not reveal any significance on patients’ outcomes.

TREM-1 and CD68 infiltration are also reciprocally correlated, however, none of the two markers is significantly associated to CD4 and CD8 infiltration. These points may configure a gap and deserve a comment: in a retrospective study on a database of 7731 cases, a high frequency (30.2%) of clinic-pathological stage discrepancy has been demonstrated in OP-SCCs [35]; in another study on 319 cases, long-term survivals in patients with advanced-stage have been also reported [36]. An interpretation of these events may be that the biological interference of HPV has influenced the relationships between tumour cells and its background: for these reasons, the pathological staging of OP-SCCs has been subjected to a radical revision in view of the last TNM edition [37], where HPV-related and non-related tumours have been separated. By contrast, the absence of a significant relation between TREM-1 and p16 expression (respectively negative and positive on survival) may be more simply explained by the relatively little number of cases in our series.

In conclusion, the very strict relation between TREM-1 expression and survival in our series of OP-SCCs needs to be further discussed towards biological and therapeutics meanings.

## Abbreviations

H-E: haematoxylin-eosin staining
HPF: high power field
HPV: human papillomavirus
HR: hazard ratios
OP-SCCs: oropharyngeal squamous cell carcinoma
OS: overall survival
TAMs: tumour-associated macrophages
TME: tumoral microenvironment
TREM-1: triggering receptor expressed on myeloid cells-1
